# The evaluation and brain representation of pleasant touch in chronic and subacute back pain

**DOI:** 10.1016/j.ynpai.2018.10.002

**Published:** 2018-10-29

**Authors:** F. Nees, K. Usai, M. Löffler, H. Flor

**Affiliations:** Department of Cognitive and Clinical Neuroscience, Central Institute of Mental Health, Medical Faculty Mannheim, Heidelberg University, Mannheim, Germany

**Keywords:** Pleasant touch, Brain, Chronic pain, Subacute pain, Chronicity

## Abstract

If touch is perceived as pleasant, it can counteract the experience of pain. However, its pain-inhibitory function might be disturbed in chronic pain and this could contribute to pain-related interference. We investigated the perception of pleasant touch and its brain correlates in chronic back pain patients (CBP) compared to subacute back pain patients (SABP) and healthy controls (HC) using soft brush strokes. CBP showed less positive evaluations of touch. We found the highest activation in somatosensory and insular cortices in CBP, ventral striatum (VS) in SABP, and the orbitofrontal cortex in HC. Brain responses were significantly positively correlated with pleasantness ratings in HC and SABP, but not CBP. Further, the insula responses in CBP were positively correlated with pain-related interference and the VS activation in SABP correlated negatively with affective distress. Brain and behavioral changes in the processing of touch and its pleasantness may be a marker of pain chronicity and raise questions about the therapeutic value of pleasant touch in pain prevention and treatment.

## Highlights

• Chronic back pain (CBP) showed less positive evaluations of touch.
• Highest response to pleasant touch in SI and SII and insula in chronic back pain.
• Highest response to pleasant touch in ventral striatum in subacute back pain (SABP).
• Correlations of brain responses with pain interference in CBP and distress in SABP.
• Brain-behavior changes in pleasant touch processing may be a marker of pain chronicity.

## 1 Introduction

Touch is an important somatosensory modality. Pleasant or painful touch representations can drive behavioral adaptations to reach homeostatic balance (Craig, 2003, Craig, 2009), which may involve a common neurobiology of pain and pleasure (Leknes and Tracey, 2008). Pleasant touch is mediated by C-tactile afferents, a class of low-threshold unmyelinated C-fibers (Olausson et al., 2002, Löken et al., 2009). In the brain, pain and pleasant touch are not only represented in the somatosensory cortices (S1 and S2), but are also encoded in circuits involved in cognitive and emotional-motivational processes, including the modulation of pain by emotions (Kamping et al., 2013). These circuits include the orbitofrontal cortex (OFC), the anterior cingulate cortex (ACC), the ventral striatum (VS), and the insular cortex (e.g., Baliki et al., 2012, Björnsdotter et al., 2010, Craig, 2009, Davidovic et al., 2017, Hashmi et al., 2013, Lamm et al., 2015, May et al., 2014, McGlone et al., 2012, Morrison et al., 2011, Olausson et al., 2002, Rolls, 1999, Rolls, 2010, Sailer et al., 2016, Vachon-Presseau et al., 2016). The insula is pivotal for the emotional components of sensory processes, including pain and pleasant touch (McGlone et al., 2012), and is involved in empathy for pain (e.g., Singer et al., 2004). There is also an overlap in the striatum, which is activated during reward processing and also involved in the prediction of pain chronicity (e.g., Baliki et al., 2010).

Pleasant touch may act as a positive reinforcer that could have beneficial pain-reducing effects. Touching the body area affected by illness can enhance psychological and physical functioning, induce stress reduction and pain relief, and enhance coping abilities and general health ratings (e.g., Ventegodt et al., 2004, Weze et al., 2005). This is also in line with findings in chronic pain patients, who show a shift to enhanced aversive processing of pain-related stimuli (Hashmi et al., 2013), and an alteration in the processing of appetitive stimuli. For example, low back pain patients attributed less hedonic value to food than controls (Geha et al., 2014), and fibromyalgia patients showed an inability to inhibit experimental pain during the presentation of positive pictures (Kamping et al., 2013).

The perception and processing of pleasant touch might likewise be altered in the chronic state. Moreover, this might be different already in the early state of the disorder in subacute patients. We examined the brain responses to touch stimulation and ratings of pleasantness during functional magnetic resonance imaging (fMRI) in chronic (CBP) and subacute patients (SABP) with back pain as well as healthy control individuals (HC). Since pain-related interference with different areas of life and pain-related affective distress are core variables in cognitive-behavioral conceptualizations of chronic pain (e.g., Turk et al., 2003, Turk and Flor, 2013), we also examined to what extent they were related to the processing of pleasant touch.

## 2 Materials and methods

### 2.1 Participants

We investigated CBP (N = 20; mean age = 46.25; 9 females), SABP (N = 19; mean age = 45.37; 10 females), and HC (N = 30; mean age = 40.23; 16 females), matched for education (see Table 1 for sample description).

**Table 1:** Characteristics of the study populations.

	Chronic back pain	Subacute back pain	Healthy controls	Group comparisons significance
Number	20	19	30	
Age, years; mean (SD)	46.25 (13.65)	45.37 (14.64)	40.23 (15.63)	n.s.
Sex female/male; number	9/11	10/9	16/14	n.s.
Medication use (N)	Blood pressure regulation (2); pain treatment (ibuprofen, aspirin, 6), treatment of depressive symptoms (trimipramin, 1)	Pain treatment (ibuprofen, aspirin, 2)	–	
Education, years; median (range) West Haven-Yale Multidimensional Pain Inventory; sum (SD)	13.28 (9–17)	13.49 (9–16)	13.88 (8–16)	n.s.
Pain Intensity	2.74 (1.57)	2.47 (1.83)	1.29 (2.01)	p < 0.05*
Pain-related Interference	3.1 (1.84)	3.225 (1.98)	2.61 (2.45)	p < 0.05*
Affective Distress	3.12 (1.88)	2.15 (1.88)	2.25 (1.68)	p < 0.05*

SD = standard deviation; n.s. = non-significant; *significant differences between chronic back pain patients/subacute back pain patients and healthy controls.

Inclusion criteria for the CBP were: pain localized to the upper or lower back, minimum frequency 3 times/week, more than 6 months duration. As treatment recommendations for chronic pain patients indicate pharmacotherapy, we did not exclude patients with medication. Previous medication and dose of medication as well as comorbidity were carefully assessed and used as covariates in the analyses.

Inclusion criteria for subacute back pain were: pain localized in the upper or lower back or both, duration of pain between 7 and 12 weeks, as previously suggested (Dionne et al., 2008, Chanda et al., 2011). We also included individuals with several short pain episodes that did not exceed 3 months in the past.

Exclusion criteria for all three samples were: age <18 or over 70 years, neurological complications, psychotic episodes, current drug abuse, left-handedness, major illness, pregnancy, a pacemaker or metal parts in the body.

The participants underwent psychometric assessments, followed by a touch stimulation procedure during fMRI.

### 2.2 Psychometric assessment

All participants underwent the Structured Clinical Interview for DSM-IV (SCID-I; German version; Wittchen et al., 1997) and were tested for both axis I and axis II mental disorders. Pain characteristics and pain-related antecedents and consequences were assessed via a structured pain interview (Flor and Turk, 2011). The West Haven-Yale Multidimensional Pain Inventory (MPI; German version; Flor et al., 1990) was employed to assess pain intensity, pain-related interference, affective distress, social support, life control as well as spouse responses to pain and the patients’ activity levels.

### 2.3 Touch stimulation

For the application of touch, we used an MR-compatible robotic tactile stimulator (cf., Essick et al., 2010) to deliver controlled slow soft brush strokes to the left forearm, which was comfortably stabilized. The stroking parameters were: 60 mm wide brush of fine, brush end centered approximately 2 cm above the skin, velocity of 3 cm/s, force calibrated to 0.4 N ± 0.05, stroking delivered in a proximal-to-distal direction with a duration of 3 s for each stroking stimulus. These stroking stimuli have been shown to be perceived as pleasant (Essick et al., 2010).

*Design.* The touch stimulation procedure was part of a learning experiment, where the touch stimuli were applied in the habituation phase at the beginning of the experiment. The participants received 6 touch stimuli during fMRI, separated by an inter-trial interval between 8 and 12 s (total duration of the stimulation session, including pleasantness ratings, see below, was 6 min). We used a short stimulation sequence that reached the necessary minimum of stimulation events needed for reliable and valid summation of the touch trials (N = 6), but avoided the development of allodynia symptoms in the pain samples (Finnerup et al., 2010, Maier et al., 2010). This was done to ensure a positive, not pain-related quality of the touch stimulus for all participants.

Pleasantness ratings. After the stimulation procedure, the participants rated the touch stimuli using the valence scale of the Self-Assessment Manikin (SAM; Bradley and Lang, 1994), which uses non-verbal descriptors that were converted into a scale ranging from 1 (“very unpleasant“) to 9 (“very pleasant“).

*Magnetic resonance imaging.* Magnetic resonance imaging was performed on a 3 Tesla Tim TRIO whole body scanner (SIEMENS Healthineers, Erlangen, Germany), equipped with a 12-channel head coil. Shimming of the scanner was done to account for maximum magnetic field homogeneity and a standard gradient field map was recorded at the beginning of each measurement. For the functional protocol, 40 contiguous axial slices (slice thickness: 2.3 mm, slice gap: 0.7 mm) were acquired using a T_2_*-weighted gradient-echo echo-planar imaging (EPI) sequence with GRAPPA technique (acceleration factor 2, repetition time (TR) = 2350 ms, echo time (TE) = 22 ms, matrix size = 96 × 96, field of view (FoV)= 220 × 220 mm^2^, flip angle (α)=90°, bandwidth (BW)=1270 Hz/px). For structural reference, we used a T_1_-weighted magnetization prepared rapid gradient echo (MPRAGE) sequence (TR = 2300 ms, TE = 2.98 ms, matrix size = 240 × 256, field of view (FoV) = 240 × 256mm^2^, flip angle (α) = 9°, bandwidth (BW) = 240 Hz/px) recording 192 sagittal slices.

### 2.4 Data processing and statistical evaluation

*Magnetic resonance imaging* The analyses of the fMRI data were performed using Statistical Parametric Mapping software (SPM12 (v6685), Wellcome Trust Centre for Neuroimaging, Institute of Neurology, University College London, UK), implemented on MATLAB R2016a (The MathWorks Inc., Natick, MA, USA). The first three scans were excluded from the analyses to account for T_1_-saturation effects. To reduce geometric distortions and improve spatial accuracy, gradient field map correction was performed on the remaining EPI images using the FieldMap toolbox in SPM. Afterwards the scans were realigned to the fourth image of the session using a rigid body transformation. A mean image was created, and the realigned and unwarped images were corrected for differences in acquisition time. The mean image was then co-registered to the T_1_ structural image, before the anatomical image was normalized into a standard stereotactic space (MNI – Montreal Neurological Institute, Quebec, Canada) using tissue probability maps in SPM12. The nonlinear transformation parameters were then applied to the functional images. Finally, the data were smoothed with a 7 mm^3^ (full width half maximum) Gaussian kernel. Event-related blood oxygenation level-dependent (BOLD) responses were convolved with a canonical hemodynamic response function. Each subject’s data set was high-pass filtered (temporal cut-off: 128 s) to remove low-frequency drifts and corrected for serial autocorrelations using first-order autoregressive functions AR(1).

We modeled the touch stimulation block and included subject-specific regressors with events at the time of touch stimulus onset and of inter-trial interval onset (i.e. the baseline), respectively. As regressors of no interest, we included the six scan-to-scan motion parameters derived from the affine part of the realignment procedure to account for residual movement effects. The individual contrast (stimulation versus baseline) images were subsequently included in a second level random effects analysis using the full factorial model of SPM12. The problem of non-independent data within subjects as well as error variance heterogeneity was addressed by performing a non-sphericity correction. After calculating fixed effect analyses for each subject, second level random effects analyses were performed for the contrasts using analysis of variance (ANOVA) with group as a between factor. T-tests were calculated for significant main effects based on whole brain and small volume correction (SVC). Based on the established literature on pleasant touch representation in healthy individuals (e.g., Rolls, 1999, Rolls, 2010), we employed the following hypothesized regions of interest (ROIs): OFC, insula, VS, ACC, amygdala, S1, S2. We chose a significance level of p < 0.05 (family wise-error (FWE) corrected; peak-level).

For graphical demonstration of the results we plotted the correlation of brain responses with the pain intensity scores and extracted weighted mean beta values of the significant brain regions (masks of the regions were used from the Wake Forest University Pick Atlas v3.0.3). Psychometric and rating data were analyzed with ANOVAs using the Statistical Package for Social Sciences (SPSS) version 15.0 for Windows with pain status (HC, CBP, SABP) as between subject factor.

## 3 Results

### 3.1 Pleasantness ratings to touch stimulation

**Fig. 1:**
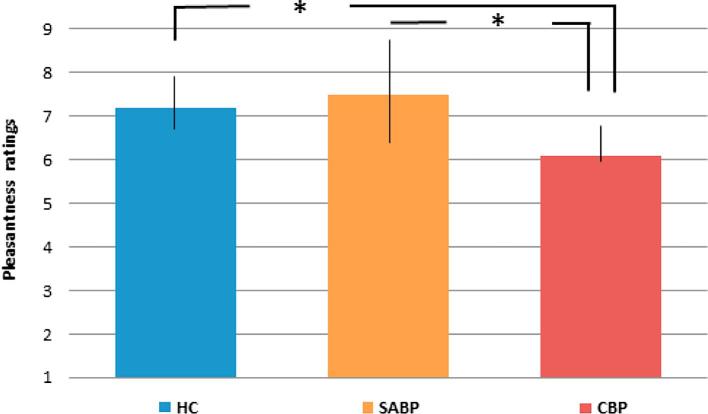
Pleasantness ratings of touch presentation in healthy controls (HC), chronic back pain (CBP) and patients with subacute (SABP).

All groups rated the touch stimulation as pleasant (ratings of 5 and above), however, the CBP group rated the stimulus as significantly less pleasant than the HC (t(2) = 2.49; p = 0.048) and the SABP (t(2) = 2.63; p = 0.049). The SABP group showed the highest, although not statistically significant, variance in pleasantness ratings, (see Fig. 1).

### 3.2 Brain responses to pleasant touch and correlations with pleasantness ratings

**Fig. 2:**
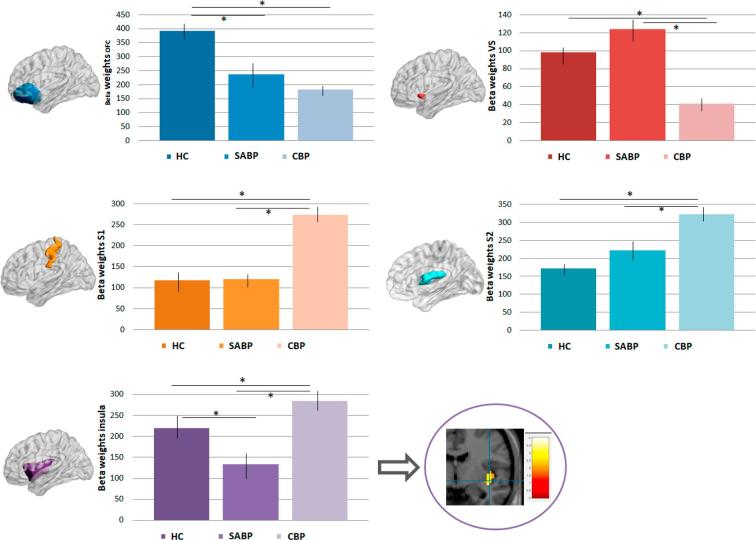
Brain responses (extracted beta weights, with standard errors) to pleasant touch, which were significantly different in healthy controls (HC), patients with chronic (CBP) and subacute back pain (SABP). *p < 0.05, ACC = anterior cingulate cortex, OFC = orbitofrontal cortex, VS = ventral striatum, S1 = primary somatosensory cortex, S2 = secondary somatosensory cortex.

We found a significant main effect of pain status (HC, SABP, CBP) for OFC (x = 18, y = 21, z = −18; t = 3.35; p = 0.031), insula (x = 36, y = −17, z = −2; t = 2.89; p = 0.043), S1 (x = 33, y = −34, z = 63; t = 3.24; p = 0.035), and S2 (x = 4, y = –32, z = 26; t = 3.32; p = 0.038), and VS (x = 12, y = 12, z = −8; t = 2.67; p = 0.041). Follow-up t-tests between the groups revealed that the HC showed significantly higher responses in the OFC compared to CBP (p = 0.041) and SABP (p = 0.045). The CBP group had a significantly higher response in the insula (compared to HC: p = 0.032, compared to SABP: p = 0.035), the S1 (compared to HC: p = 0.032; compared to SABP: p = 0.031) and S2 (compared to HC: p = 0.043; compared to SABP: p = 0.045), and reduced response in the the VS compared to SABP (p = 0.038) (see Fig. 2 and also Table 2 for further whole brain differences between the groups).

**Table 2:** Significant group-related brain activations to pleasant touch.

Brain structure	MNI coordinates			*T*_max_	*Cluster (voxels)*	*p_FWE_*
	*x*	*y*	*z*			
Inferior parietal cortex	50	−51	40	3.97	45	0.039
Medial occipital cortex	−3	−77	6	3.90	30	0.028
Inferior frontal gyrus	43	49	−10	3.93	21	0.030
Orbitofrontal cortex^a^	18	21	−18	3.35	38	0.031
Somatosensory cortex 1^a^	33	−34	63	2.89	193	0.043
Somatosensory cortex 2^a^	4	–32	26	3.32	186	0.038
Ventral striatum^a^	12	12	−8	2.67	23	0.041
Insula^a^	36	−17	−2	3.35	19	0.031

a Based on small volume corrected region of interest analysis.

We found no significant effects for the ACC and amygdala.

We did not observe any significant associations between the pleasant touch ratings and the brain response patterns across groups, but a positive correlation in the HC (OFC (r = 0.371), insula (r = 0.376), S1 (r = 0.159), S2 (r = 0.488), VS (r = 0.159); all p < 0.05) and SABP (OFC (r = 0.266), insula (r = 0.465), S1 (r = 0.154), S2 (r = 0.154), VS (r = 0.156); p < 0.05). The correlations were not significantly different between HC, SABP and CBP.

### 3.3 Association of brain responses to pleasant touch with pain intensity, pain-related interference and affective distress

**Fig. 3:**
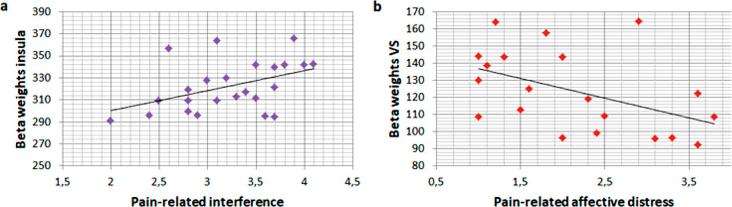
a) Correlation of insula responses during pleasant touch and pain-related interference in chronic back pain patients (CBP) and b) correlation of ventral striatal (VS) responses and affective distress in subacute back pain patients (SABP).

We observed a significant positive correlation between pain-related interference and insula responses in CBP (insula: r = 0.234, p = 0.038; see Fig. 3a), and a significant negative correlation between VS responses and affective distress scores in SABP (r = −0.398, p = 0.033; see Fig. 3b).

There were no significant associations between brain responses and pain intensity scores, neither across nor within the groups.

## 4 Discussion

Until now, there has been little experimental evidence on the evaluation and representation of pleasant touch in relation to chronic and subacute pain. We showed that the brain is differentially engaged in the processing of pleasant touch in these different pain states. All three groups (CBP, SABP, HC) showed touch-related activation in brain regions previously identified as significantly involved in the processing of pleasant touch, including the OFC, insula, VS, ACC, S1 and S2, and also frontal areas. However, except for the ACC and amygdala, the response magnitudes of these regions differed significantly between HC, CBP and SABP. These differences were also apparent in the valence of pleasant touch, with the CBP perceiving the touch stimulation as significantly less pleasant than the HC and SABP. Moreover, we observed a significant association of insula responses and pain-related interference in CBP and VS responses and pain-related affective distress in SABP.

### 4.1 Brain activation in CBP: Deficient processing of touch and its pleasantness

In line with Case et al. (2016), we found decreased pleasantness ratings of touch stimuli in the CBP group. This was mirrored by increased responses to pleasant touch in the S1 and S2 cortex compared to both HC and SABP. These regions are involved in the sensory processing of pain and habituation processes related to pain. The ability to habituate to pain may represent an important protective mechanism. Reduced pain perception over time, following repetitive painful stimulation, was reflected in a decrease in activation in brain regions like S2, insula or striatum (putamen) (Bingel et al., 2007). The higher activation in S1 and S2 might reflect enhanced sensory and reduced affective processing of the touch stimulation and thus be indicative of an impaired modulation of pain by pleasant stimuli. Moreover, in contrast to the OFC as a correlate of the affective aspects of touch, the somatosensory cortex has been more strongly implicated in the representation of neutral touch stimulation (Rolls et al., 2003), further reinforcing the notion that pleasant touch processing is altered in a more neutral direction in the CBP. This is also supported by the higher response of the posterior insula in the CBP compared to HC and SABP. This part of the insula has been related to interoceptive awareness (Kuehn et al., 2016). The higher activation of the insula in CBP might thus impair sufficient or successful processing of pleasant touch inducing a hyper-processing of sensory rather than affective aspects of pleasant touch as well as a stronger focus on the current own pain as an interoceptive dominant process in chronic pain. CBP might thus have a reduced capacity to benefit from exposure to pleasant stimuli (cf. Kamping et al., 2013), also with respect to the healing power of pleasant touch such as provided in massage therapy, which is important to consider in treatment. In addition, CBP may show a persistent focus on negative stimuli and events, including their own pain. This is in line with the lower valence ratings related to pleasant touch, which were not significantly related to the brain response patterns in CBP. These results add to the findings of stronger activation of brain circuits mediating aversive states in CBP (Hashmi et al., 2013). Future studies need to directly assess the effects of touch on pain and should vary the length of the stimulation protocol under different pain versus pain-free conditions. In our study, we have used a very short stimulation protocol, based on the motivation to avoid any possible development of sensitization in the patient samples and thus to achieve an unbiased assessment of the response to a touch stimulus.

CBP showed the lowest VS responsivity to pleasant touch, which was significantly different from SABP, but not HC. While the insula may be involved in the general emotional processing of stimuli (e.g., Uvänas-Moberg et al., 2005), the VS may code more specific aspects of rewarding stimuli (for review see Robbins and Everitt, 1996). These functions involve the coding hedonic aspects of touch (May et al., 2014) and the prediction of future outcome, which has also been observed in the context of pain and analgesia (Baliki et al., 2010, Zubieta and Stohler, 2009). Striatal-prefrontal connectivity has been found as a predictor of chronicity, in line with higher striatal activation in the subacute group in this study. Although not directly associated with VS responses, we also observed higher pleasantness ratings of the touch stimuli in SABP compared to CBP. Thus, SABP might still be able to feel pleasurable touch, while the perception of touch as pleasant may be impaired in a chronic state. This is also mirrored in the correlations between brain responses and pleasantness ratings that were significant in SABP, but not in CBP. Our data suggest a stronger processing of pleasurable events in SABP compared to CBP, where deficient processing occurs. This is in line with operant pain models that suggest that enhanced responsivity to reward might be an important factor in pain chronicity (Main et al., 2014), with finding that the transition stage from acute to chronic pain shows enhanced involvement of reward-related brain activations (Baliki et al., 2012) and with the predictive role of striatal responding for pain symptoms in adolescents (Nees et al., 2017).

### 4.2 Brain activation in SABP

The SABP group showed a significantly lower responsivity to pleasant touch in the posterior insula compared to HC and CBP. Previous studies documented an involvement of the insular cortex in the representation of pleasant touch (Rolls et al., 2003, Davidovic et al., 2017), with the posterior insula coding not only painful touch but also pleasant touch with a strong connectivity to the anterior insula and thus brain areas involved in emotional processing (Davidovic et al., 2017). The activation peak in our study was found in the posterior insula. The posterior insula has been shown to be related more to sensory-discriminative than emotional aspects of pain processing (e.g., Albanese et al., 2007, Bingel et al., 2007, Oertel et al., 2012, Wiech et al., 2010). Our findings could relate to compensatory processes in the subacute phase, with a reduction of sensory processing in favor of emotional processing as an early indicator of the altered representation of touch in the chronicity process. Although this differential pattern between CBP and SABP (with CBP showing increased, but SABP reduced insula responses to pleasant touch) cannot be easily explained, such “inverted” patterns have also previously been observed in individuals, who are in a disorder-specific transition period, like traumatized individuals who have not (yet) developed posttraumatic stress disorder (van der Werff et al., 2013). Moreover, a shift in the processing of painful stimulation from nociceptive brain areas to emotion-related circuits in the transition from subacute to chronic pain has also been described by Hashmi et al. (2013). In line with these findings, SABP compared to CBP (but not HC) showed increased responses in the VS. As noted above, the intact pleasantness ratings of the touch stimuli in SABP and the significant correlations between brain responses and pleasantness ratings suggest that SABP might still be able to feel pleasurable touch although their brain activation patterns are already altered.

### 4.3 Brain changes in both SABP and CBP

The OFC has been implicated in the processing of affective touch (e.g., Olausson et al., 2002, Rolls et al., 2003), and the processing of various rewarding stimuli such as taste (Kringelbach et al., 2003, Veldhuizen et al., 2010), erotic stimuli (e.g., Sescousse et al., 2010), odors (e.g., Rolls, 2010), and money (e.g., O'Doherty et al., 2001). In the context of pain and reward, this region was found to trigger acute pain inhibition by the presentation of a reward (Becker et al., 2017). OFC responsivity might play a critical role in the interaction of pain and pleasure and related emotional modulations. In the present study, we observed increased OFC responses in HC compared to SABP and CBP. Some pain inhibitory circuits might thus be disturbed already at the level of subacute back pain, and a reduced capacity for pain suppression might be present. This is also in line with studies on conditioned pain modulation, where pain ratings and pain-evoked potentials are attenuated, and a higher response in the OFC during this pain suppressive effect has been observed (e.g., Moont et al., 2011).

### 4.4 Limitations

The present study needs to be considered in the light of some limitations. We did not perform control stimulation (e.g. static touch or vibration) to differentiate the role of the affective component from touch stimulation per se. However, we analyzed the linear relationships between brain responses and affective pleasantness ratings of the touch stimulation within and across groups, which allowed us to directly relate valence and brain activation to the touch stimulation. We also do not know who in the SABP group will develop chronic pain and who will be resilient and how the group differences we found relate to the final outcome on pain chronicity. This will require longitudinal studies. A further limitation of our study is that we covaried out the use of medication. Our sample was too small to include past and current medication use as a separate factor that could explain additional variance in the response to somatosensory and hedonic stimuli. Last, in our experimental design we used 6 stimuli of pleasant touch, applied in the habituation phase at the beginning of a respondent conditioning experiment. Although this short protocol provided significant information, and avoided the occurrence of potential allodynia in the pain samples, a longer protocol with additional somatosensory and hedonic controls might have increased the power and clarified the specificity of the touch effects.

### 4.5 Summary

Our data highlight a deficient processing of pleasant touch in CBP and alterations of the response also in the subacute state. These findings suggest that changes in the representation of pleasant touch could be a marker of deficient affective processing in the transition from acute to chronic pain. To what extent the group differences in the processing of pleasant touch extend to other types of somatosensory or hedonic stimuli needs to be determined in future studies. Moreover, our findings can inform longitudinal studies on pleasure and pain and could contribute to the identification of mechanisms predicting or preventing chronicity as well as to the development of therapeutic targets. The neural and perceptual processing of pleasant touch has been a relatively neglected topic in pain research. The possibility to precisely apply brush stimuli in an automated fashion with well defined neural and perceptual responses suggests an inclusion of pleasant touch in quantitative sensory testing as well as brain imaging protocols in pain research*.*
